# Migration behaviour of 2 clinically excellent cementless stems with different design rationales: 5-year follow-up of a randomised RSA-study

**DOI:** 10.1177/1120700021995482

**Published:** 2021-02-17

**Authors:** Paul van der Voort, Danny van Delft, Edward R Valstar, Bart L Kaptein, Marta Fiocco, Rob GHH Nelissen

**Affiliations:** 1 Department of Orthopaedics, Biomechanics and Imaging Group, Leiden University Medical Center, Leiden, The Netherlands; 2 Department of Biomechanical Engineering, Faculty of Mechanical, Maritime and Materials Engineering, Delft University of Technology Delft, The Netherlands; 3 Department of Medical Statistics and Bioinformatics, Leiden University Medical Center, Leiden, The Netherlands; 4 Mathematical Institute, Leiden University, Leiden, The Netherlands

**Keywords:** Randomised clinical trial, Roentgen stereophotogrammetric analysis, total hip arthroplasty

## Abstract

**Introduction:**

Excellent long-term survival has been reported for both the Taperloc and the Mallory-Head cementless stems. However, little is known about the migration behaviour of these stems which have different design rationales. The purpose of this randomised clinical trial was to compare the migration and clinical outcomes of these stems during 5 years of follow-up.

**Methods:**

42 consecutive hips in 38 patients scheduled to receive cementless THA were randomised to either a Taperloc or a Mallory-Head stem. Evaluation took place preoperatively and postoperatively on the second day, at 6, 12, 26, and 52 weeks, and annually thereafter. Primary outcome was stem migration measured using roentgen stereophotogrammetric analysis (RSA) and secondary outcomes were the Harris Hip Score (HHS) and 36-Item Short-Form Health Survey (SF-36). No patients were lost to follow-up; in 1 patient the THA was removed due to deep infection 3 months postoperatively. In 6 hips migration measurements were not possible due to insufficient marker configuration.

**Results:**

Throughout the follow-up period of 5 years, 3-dimensional migration was comparable between the Taperloc and the Mallory-Head stems (*p*-values > 0.05). However, at the 5-year follow-up point the retroversion of the Mallory-Head stem was 0.9° more than the Taperloc stem (*p* = 0.04). Initial subsidence and retroversion were respectively as large as 6.8 mm and 3.6° for the Taperloc stem and 5 mm and 3.6° for the Mallory-Head stem. After the first postoperative year, both implants had stabilised. The mean increment of HHS, as well as the SF-36 scores during the 5-year follow-up, were comparable between the 2 stems.

**Conclusions:**

The excellent long-term survival of both designs was confirmed in this study showing comparable initial migration with subsequent stabilisation. However, the Taperloc design with a flat, wedged geometry showed better rotational stability.

## Introduction

**Figure 1.**
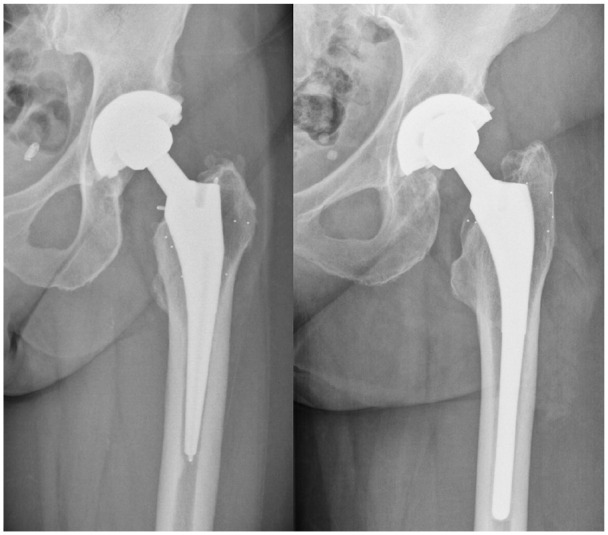
Radiographs of the Taperloc femoral stem on the left and the Mallory-Head femoral stem on the right.

The stems of the Taperloc and Mallory-Head total hip arthroplasty (THA) are straight and tapered designs, achieving metaphyseal fixation through a porous coating. The Taperloc stem has a rectangular, flat and thin transverse geometry, while the Mallory-Head stem has a circular transverse geometry (Figure 1).^1^

These 2 stems with different design rationales have proven to be safe choices in THA, showing excellent survival in long-term studies.^2,3^ However, studies evaluating the migration behaviour, as measured with roentgen stereophotogrammetric analysis (RSA), of these clinically well performing stems are scarce and non-existent for the Mallory-Head stem.^4–7^ The association between short-term RSA results and future loosening of THA has been described for different cemented THA designs.^8,9^ However, the influence of particular design features on the migration behaviour of cementless stems is rarely described.

In this study, we report the 5-year results of a randomised trial in which we compare the migration, measured with RSA, and clinical outcome of the 2 differently designed cementless stems, thereby analysing the influence of particular design features on migration behaviour. We hypothesised that the migration and clinical outcome of the Taperloc stem would be comparable with that of the Mallory-Head stem.

## Patients and methods

### Study design

After the approval of the institutional medical ethical board was obtained (reference code P00.167), all consecutive patients scheduled to receive a cementless primary THA for symptomatic osteoarthritis, either primary or secondary to a systematic inflammatory disease, were approached for participation in a randomised, clinical RSA study. Included patients gave their written informed consent and were randomised to receive either a cementless Taperloc (Biomet, Warsaw, IN, USA) or a cementless Mallory-Head (Biomet, Warsaw, IN) stem. Treatment allocation was randomised with use of a computer-generated randomisation scheme and bilateral cases were allowed. The study design was single-blinded; patients were unaware of the allocated stem, but surgeons who implanted the stem and clinical observers evaluating the radiographs could not be blinded. The study was performed in compliance with the Helsinki Declaration. Reporting of the trial was in accordance with the Consolidated Standards of Reporting Trials (CONSORT) and the ISO standard [Implants for surgery - Roentgen stereophotogrammetric analysis for the assessment of migration of orthopaedic implants (16087:2013)].^10,11^

### Surgical technique

All THAs were implanted by experienced specialist hip surgeons, or under their direct supervision, through a lateral approach in the lateral decubitus position. For RSA measurements, 3–8 1-mm tantalum beads were inserted into the proximal femur during surgery. All patients received the same rehabilitation programme commencing with passive and controlled active movement on the first postoperative day and mobilisation with full weight bearing was started on the second postoperative day.

### Implants

The Taperloc stem has a rectangular cross-sectional geometry with a single taper wedge design. The stem is made of a titanium alloy (Ti-6A1-4V), with a porous plasma-sprayed coating on the proximal third, and a smooth surface on the middle and distal third. The Mallory-Head Porous stem has a round cross-sectional geometry with a dual-tapered design. The stem is characterised by an anterior and posterior flange and wide lateral fin. The stem is made of a titanium alloy (Ti-6A1-4V), with a porous plasma-sprayed coating on the proximal third, a grit-blasted surface on the middle third, and a smooth satin-textured surface on the distal third. Neither of the porous surface of either stem used in this trial was augmented with a coating. All patients received a 28-mm cobalt-chrome head and a cementless Mallory-Head finned Ringloc acetabular cup (Biomet, Warsaw, IN).

### Follow-up

Patients were evaluated preoperatively and postoperatively at 6 weeks, 3 months, 6 months, 1 year, and annually thereafter, until 5 years of follow-up. At each evaluation, RSA radiographs were obtained and the Harris Hip Score (HSS) and 36-Item Short-Form Health Survey (SF-36) were determined.^12,13^ Conventional anteroposterior and lateral radiographs were acquired preoperatively, at 6 weeks, 2 years, 5 years and on indication (e.g. pain or suspected failure). On the preoperative radiographs the metaphyseal canal shape was classified by the canal flare index (CFI), defined as ratio of the intra-cortical width of the femur at a point 20 mm proximal to the lesser trochanter and at the diaphyseal canal isthmus.^14^ A CFI of <3.0 described a stovepipe shape, 3.0–4.7 was normal, and 4.7–6.5 described a champagne-flute shape.^15^ On the 6-week postoperative radiographs the stem orientation (i.e. varus, neutral or valgus) was determined. The 2- and 5-year postoperative radiographs were evaluated for presence of radiolucent lines,^16^ bone resorption, cortical thickening and pedestal formation.

### RSA technique

RSA radiographs were obtained using a uniplanar setup with the patient in supine position and the calibration cage (Carbon box, Leiden, The Netherlands) underneath the examination table. The first RSA examination was made before weight-bearing on the second postoperative day and the relative position of the stem to the bone at that time served as the baseline for all further examinations. A marker-based analysis was carried out to calculate migration over time (Model-Based RSA software, version 3.34; RSA*core*, The Netherlands), using 4 stem markers: 3 markers were attached to the stem by the implant manufacturer, and the centre of the head acted as a fourth marker. Migration was expressed as translations along and rotation about 3 axes (longitudinal, transverse, and sagittal) of a right-handed orthogonal coordinate system. Since the failure mechanism of stems consists of subsidence and retroversion,^8^ the primary effect variables were translation along and rotation about the longitudinal axis. The accuracy of RSA measurements was determined by obtaining double examinations of 19 stems 1 year postoperatively. Assuming zero migration in the brief time interval between these double examinations, the limits of the 95% prediction interval of accuracy of zero migration were determined (Table 1).^17^ For all examinations, the mean error of rigid body fitting of the RSA markers in the femur was below 0.35 mm; the mean condition number of the RSA markers was 29 (standard deviation [SD] 16; range 11–90) in the femur. Bone markers were defined as unstable when they moved more than 0.5 mm with respect to the other bone markers. Unstable markers were excluded from the analyses. These values satisfy the marker stability and distribution criteria of the ISO guideline; (ISO 16087:2013).^11^

**Table 1.** Accuracy of RSA measurement (upper limits of 95% zero motion confidence interval).

Stem	Transverse	Longitudinal	Sagittal
	(x-axis)	(y-axis)	(z-axis)
Translation (mm)	0.07	0.10	0.35
Rotation (°)	0.42	0.53	0.20

### Statistical analysis

Based on earlier RSA studies and owing to the high degree of accuracy of RSA, 20 stems were required for each trial arm, as was standard at our institution at the time this study was designed.^8,18–20^ The distribution of the acquired data was tested for normality using the Shapiro-Wilk test. Normality was assumed if the test statistic W was >0.90. Measured values of normally distributed data are reported as the mean and the SD; measured values of non-normally distributed data are reported as the median and the range. Estimates are reported as the mean and the 95% confidence interval (CI). Reported analyses were performed according to the per-protocol principle to reflect the genuine effect of treatment (i.e. Taperloc or Mallory-Head). To safeguard for attrition bias, all analyses were repeated according to the intention-to-treat principle and compared with the outcomes of the per-protocol analyses.

Migration and increase in HHS throughout the follow-up period were analysed with use of a linear mixed model (LMM) with subject as a random effect. This model deals effectively with repeated measurements, missing values and variation in duration of follow-up.^21^ Differences between the stems were assessed by estimating the main treatment effect and the ‘stem type’ × ‘time interaction’, both as an overall effect over the entire follow-up period taking the repeating measurements into account. The assessment of the interaction term allows for the investigation of possible time-varying mean differences. At the 2- and 5-year follow-up, the mean differences were assessed with the use of an unpaired Student’s *t-*test as specified in the study protocol. As a sensitivity analysis, separate adjusted analyses were carried out with age, gender, body mass index (BMI), and diagnosis (primary or secondary osteoarthritis) as covariates. SF-36 scores were compared with the use of an unpaired Student’s *t*-test (normally distributed data) or Mann-Whitney U-test (MWU, non-normally distributed data). A *p*-value of <0.05 was considered to be significant (SPSS version 20.0; SPSS, Chicago, IL).

## Results

### Patients

**Figure 2.**
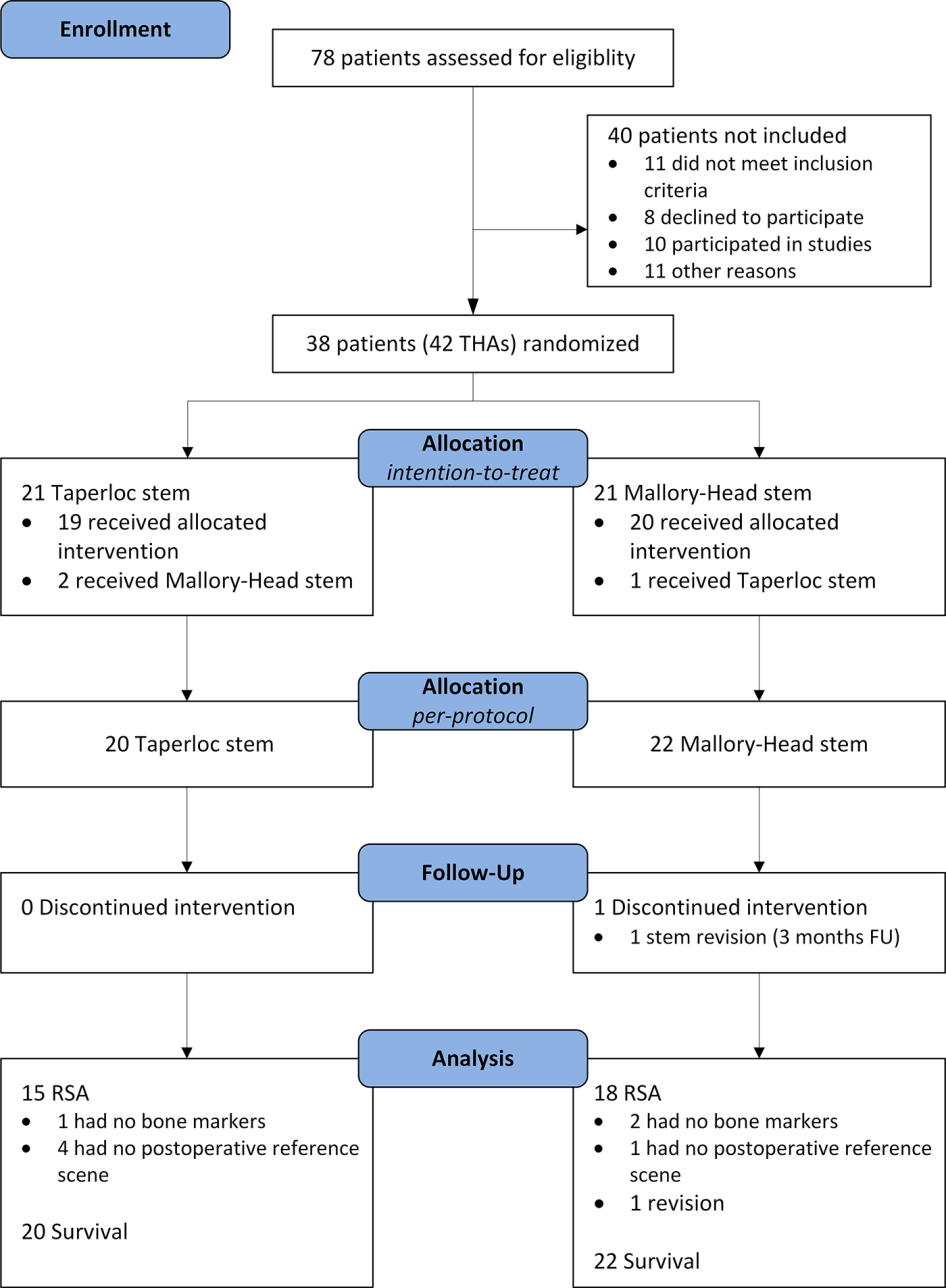
CONSORT flowchart of patient recruitment, allocation and follow-up. THA, total hip arthroplasty; FU, follow-up.

A total of 88 consecutive THAs in 78 patients were assessed for inclusion and 42 THAs in 38 patients were randomised (Figure 2). 19 patients (20 THAs) received a Taperloc stem and 20 patients (22 THAs) received a Mallory-Head stem (Table 2). No patients died during the 5-year follow-up and no patients were lost to follow-up. Patients excluded from the RSA analysis remained in the study and received routine clinical and radiographic follow-up.

**Table 2.** Group characteristics at baseline.

Characteristic	Taperloc stem	Mallory-Head stem
	(*n* = 20)	(*n* = 22)
Gender *n* (%)		
Male	5 (25%)	9 (37.5%)
Female	15 (75%)	13 (62.5%)
BMI^a^ (kg/m²)	28.6 ± 4.6	26.6 ± 5.0
Age at surgery^a^ (years)	54.7 ± 7.4	56.4 ± 7.9
Diagnosis *n* (%)		
Osteoarthritis	7 (35%)	10 (45.5%)
Rheumatoid arthritis	2 (10%)	4 (18.2%)
Osteonecrosis	5 (25%)	3 (13.6%)
Hip dysplasia	2 (10%)	3 (13.6%)
Other	4 (20%)	2 (9%)
Side *n* (%)		
Left	8 (40%)	10 (45.5%)
Right	12 (60%)	12 (54.5%)
Surgeon *n* (%)		
Consultant	16 (80%)	19 (86.4%)
Resident	4 (20%)	3 (13.6%)
Stem orientation *n* (%)		
Varus	0	0
Neutral (<3°)	19 (95%)	19 (86.4%)
Valgus	1 (5%)	3 (13.6%)
Canal Flair Index^b^	3.7 ± 0.6	3.6 ± 0.7
Dorr classfication *n* (%)		
A	3 (15%)	5 (22.7%)
B	15 (75%)	17 (77.3%)
C	2 (10%)	0
Preoperative HHS^a^ min 0–max 100 points	41.9 ± 16.3	44.8 ± 14.6

a The values are given as the mean and the standard deviation.

b The ratio of the intracortical width of the femur at a point 20 mm proximal to the lesser trochanter and at the canal isthmus, and the standard deviation.

HHS, Harris Hip Score.

### Migration

**Figure 3.**
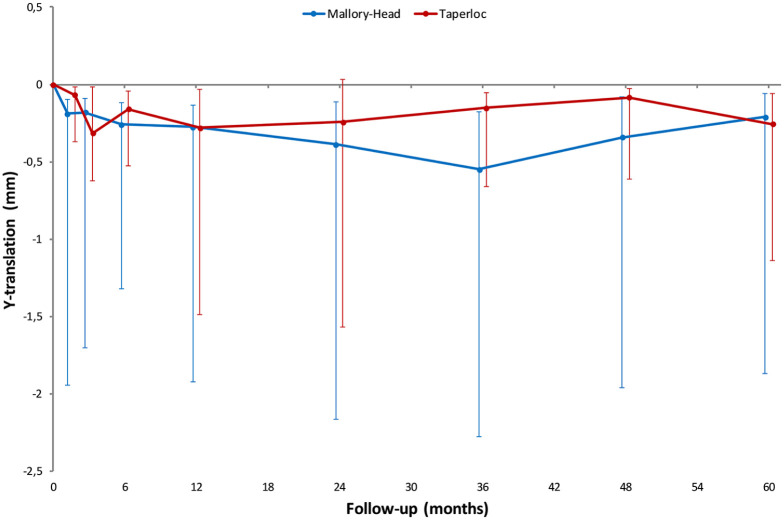
Line graphs showing the median Y-translation (i.e. translation along the longitudinal axis) with interquartile range during the 5 years of follow-up for the Taperloc and Mallory-Head stems.

**Figure 4.**
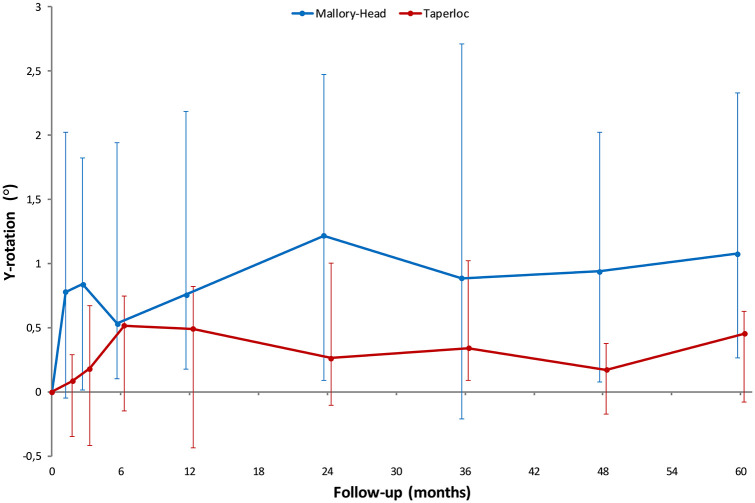
Line graphs showing the median Y-rotation (i.e. internal rotation about the longitudinal axis) with interquartile range during the 5 years of follow-up for the Taperloc and Mallory-Head stems.

Throughout the follow-up period of 5 years, the migration of the 2 femoral stem designs along and about any of the 3 orthogonal axes was not significantly different (main effect; LMM; *p*-values ⩾ 0.05; Figure 3) (Table 3). However, difference in retroversion between the 2 stems was nearly significant (main effect; LMM; *p*-value = 0.05; Figure 4) (Table 3), with the Mallory-Head stem showing more retroversion. At the pre-specified time point of 5 years postoperatively the Mallory-Head stem showed 0.9° (unpaired Student’s *t*-test; 95% CI, 0–1.8°; *p* = 0.04; Table 3) more retroversion than the Taperloc stem. There was no difference in time to stabilisation and subsequent migration; that is, no evidence of interaction. The results from the adjusted analyses were comparable with the results from the unadjusted analyses and neither age, gender, BMI, diagnosis nor CFI significantly influenced migration (LMM; *p*-values > 0.05).

**Table 3.** Analysis of femoral component migration during 5 years of follow-up.

Migration	Taperloc stem			Mallory-Head stem			PER PROTOCOL		
	*n*	Mean (SD)	Median (range)	*n*	Mean (SD)	Median (range)	Main effect	Group × time interaction	Prespecified time point
							*p*-Value	*p*-Value	*p*-Value
Translation (mm)									
x-axis (medial-lateral)									
Week 6	13	−0.05 (0.39)	0.01 (–1.2 to 0.47)	15	0.09 (0.36)	0.14 (–0.8 to 0.71)	0.53	0.88	
Month 3	13	−0.02 (0.43)	0.07 (–1.2 to 0.69)	18	0.09 (0.35)	0.08 (–0.73 to 0.82)			
Month 6	14	−0.01 (0.36)	0.05 (–1.15 to 0.31)	16	0.04 (0.34)	0.08 (–0.95 to 0.66)			
Year 1	13	0.05 (0.44)	0.11 (–1.15 to 0.81)	18	0.09 (0.42)	0.13 (–1.05 to 0.94)			
Year 2	13	0.04 (0.49)	0.13 (–1.26 to 0.8)	16	0.11 (0.51)	0.12 (–1.07 to 1.06)			0.44
Year 3	13	−0.04 (0.42)	0.09 (–1.2 to 0.45)	15	0.08 (0.45)	0.07 (–1.05 to 0.84)			
Year 4	12	0.08 (0.22)	0.12 (–0.41 to 0.48)	14	0.09 (0.38)	0.12 (–0.94 to 0.84)			
Year 5	15	0.02 (0.48)	0.11 (–1.23 to 0.89)	18	0.11 (0.38)	0.1 (–0.78 to 0.83)			0.55
y-axis (cranial-caudal)									
Week 6	13	−0.76 (1.62)	−0.07 (–4.73 to 0.28)	15	−1.27 (1.72)	−0.19 (–4.83 to 0.14)	0.75	0.23	
Month 3	13	−0.98 (1.74)	−0.32 (–5.47 to 0.22)	18	−1.14 (1.65)	−0.18 (–4.86 to 0.08)			
Month 6	14	−0.61 (1.12)	−0.16 (–4.11 to 0.01)	16	−1.08 (1.61)	−0.26 (–4.96 to 0.11)			
Year 1	13	−1.18 (2.07)	−0.28 (–6.84 to 0.23)	18	−1.25 (1.73)	−0.28 (–4.81 to 0.09)			
Year 2	13	−1.21 (2.12)	−0.24 (–7.03 to 0.32)	16	−1.34 (1.83)	−0.39 (–4.89 to 0.22)			0.80
Year 3	13	−0.72 (1.21)	−0.15 (–4.13 to 0.2)	15	−1.46 (1.85)	−0.55 (–4.88 to 0.23)			
Year 4	12	−0.41 (0.69)	−0.08 (–1.96 to 0.3)	14	−1.25 (1.78)	−0.34 (–4.93 to 0.19)			
Year 5	15	−1.13 (2)	−0.26 (–7.11 to 0.08)	18	−1.2 (1.77)	−0.21 (–4.98 to 0.21)			0.91
z-axis (anterior-posterior)									
Week 6	13	−0.14 (0.44)	−0.09 (–1.04 to 0.61)	15	−0.15 (0.47)	−0.12 (–1.05 to 0.78)	0.87	0.85	
Month 3	13	−0.15 (0.62)	−0.05 (–1.84 to 0.77)	18	−0.13 (0.44)	−0.04 (–1.06 to 0.49)			
Month 6	14	−0.09 (0.31)	−0.19 (–0.61 to 0.47)	16	−0.12 (0.45)	−0.08 (–1.13 to 0.62)			
Year 1	13	−0.28 (0.63)	−0.3 (–1.94 to 0.8)	18	−0.16 (0.47)	−0.19 (–1.1 to 0.69)			
Year 2	13	−0.2 (0.72)	−0.1 (–2.16 to 0.84)	16	−0.21 (0.57)	−0.19 (–0.96 to 0.82)			0.91
Year 3	13	−0.04 (0.42)	−0.17 (–0.64 to 1.03)	15	−0.19 (0.5)	−0.15 (–1 to 0.71)			
Year 4	12	0.01 (0.35)	−0.07 (–0.49 to 0.76)	14	−0.15 (0.38)	−0.08 (–1 to 0.67)			
Year 5	15	−0.2 (0.83)	−0.17 (–2.98 to 0.57)	18	−0.13 (0.42)	−0.19 (–0.92 to 0.8)			0.75
ROTATION (°)									
x-axis (transverse)									
Week 6	13	0.04 (0.47)	0.15 (–0.93 to 0.72)	12	−0.15 (0.47)	−0.1 (–1 to 0.54)	0.75	0.63	
Month 3	12	−0.06 (0.38)	−0.03 (–0.55 to 0.62)	14	−0.15 (0.4)	−0.22 (–0.68 to 0.56)			
Month 6	13	0.02 (0.3)	0.05 (–0.49 to 0.44)	13	−0.07 (0.5)	0.09 (–0.8 to 0.69)			
Year 1	12	−0.05 (0.47)	−0.07 (–0.79 to 0.64)	14	−0.01 (0.44)	0.05 (–0.6 to 0.73)			
Year 2	12	−0.11 (0.46)	−0.05 (–0.95 to 0.5)	12	−0.04 (0.34)	−0.05 (–0.65 to 0.48)			0.56
Year 3	12	0.11 (0.45)	0.16 (–0.59 to 0.67)	12	0.05 (0.49)	0.01 (–0.7 to 0.88)			
Year 4	11	0.07 (0.48)	−0.02 (–0.66 to 0.82)	12	0.03 (0.49)	−0.15 (–0.7 to 0.79)			
Year 5	14	−0.06 (0.5)	−0.04 (–1.02 to 0.65)	14	−0.06 (0.45)	−0.03 (–0.65 to 0.75)			0.99
y-axis (longitudinal)									
Week 6	13	0 (1.09)	0.08 (–1.59 to 2.68)	12	1.09 (1.37)	0.78 (–0.5 to 3.67)	0.06	0.97	
Month 3	12	0.23 (1.27)	0.18 (–1.89 to 3.29)	14	1.1 (1.33)	0.84 (–0.42 to 4.15)			
Month 6	13	0.38 (1.09)	0.52 (–1.45 to 3.15)	13	1.05 (1.22)	0.53 (–0.63 to 3.46)			
Year 1	12	0.29 (1.51)	0.49 (–2.54 to 3.64)	14	1.16 (1.26)	0.76 (–0.38 to 3.57)			
Year 2	12	0.33 (1.36)	0.26 (–2.94 to 2.53)	12	1.39 (1.47)	1.22 (–0.29 to 3.63)			0.10
Year 3	12	0.3 (1.16)	0.34 (–2.82 to 1.83)	12	1.23 (1.53)	0.89 (–0.51 to 3.77)			
Year 4	11	0.03 (0.98)	0.17 (–2.43 to 1.56)	12	1.16 (1.29)	0.94 (–0.33 to 3.6)			
Year 5	14	0.33 (0.96)	0.46 (–1.96 to 2.27)	14	1.26 (1.28)	1.08 (–0.49 to 3.64)			0.04
z-axis (sagittal)									
Week 6	13	0.03 (0.31)	0.02 (–0.32 to 0.66)	12	−0.05 (0.52)	−0.12 (–0.57 to 1.43)	0.31	0.62	
Month 3	12	−0.01 (0.35)	−0.02 (–0.63 to 0.58)	14	−0.13 (0.46)	−0.15 (–0.65 to 1.26)			
Month 6	13	−0.03 (0.25)	−0.02 (–0.52 to 0.45)	13	−0.12 (0.4)	−0.07 (–0.77 to 0.76)			
Year 1	12	0.04 (0.47)	−0.02 (–0.5 to 1.01)	14	−0.18 (0.36)	−0.14 (–0.75 to 0.47)			
Year 2	12	0.07 (0.58)	−0.05 (–0.63 to 1.32)	12	−0.1 (0.44)	0.01 (–0.71 to 0.81)			0.49
Year 3	12	0.05 (0.53)	0.04 (–0.75 to 1.22)	12	−0.1 (0.41)	−0.16 (–0.57 to 0.8)			
Year 4	11	0.04 (0.52)	0.09 (–0.84 to 1.22)	12	−0.07 (0.41)	−0.1 (–0.62 to 0.79)			
Year 5	14	0.03 (0.52)	0.06 (–0.98 to 0.99)	14	−0.11 (0.41)	−0.18 (–0.62 to 0.95)			0.43
Mean total point motion (mm)									
Week 6	13	1.58 (1.57)	0.99 (0.41–5.57)	12	2.61 (1.81)	1.78 (0.69–5.84)	0.18	0.15	
Month 3	12	1.71 (1.87)	1.06 (0.34–6.35)	14	2.39 (1.71)	1.72 (0.64–5.99)			
Month 6	13	1.21 (1.08)	1.04 (0.28–4.6)	13	2.32 (1.67)	1.74 (0.46–5.87)			
Year 1	12	2.15 (2.13)	1.49 (0.56–7.79)	14	2.44 (1.79)	2 (0.51–5.91)			
Year 2	12	2.15 (2.18)	1.29 (0.47–8.09)	12	2.67 (1.9)	1.84 (0.93–6.01)			0.13
Year 3	12	1.7 (1.12)	1.69 (0.42–4.39)	12	2.83 (1.76)	2.31 (0.8–5.93)			
Year 4	11	1.34 (0.67)	1.48 (0.58–2.32)	12	2.44 (1.7)	1.9 (0.73–5.75)			
Year 5	14	2.1 (2.09)	1.34 (0.37–8.22)	14	2.49 (1.77)	1.84 (0.42–6.02)			0.42

**Figure 5.**
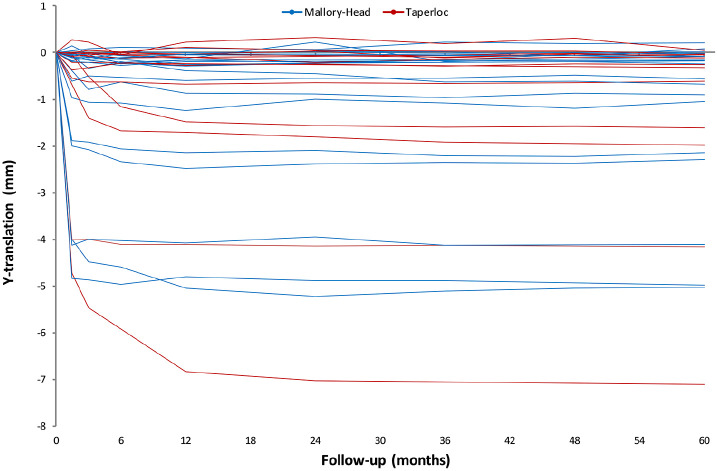
Line graphs showing the Y-translation (i.e. distal translation along the longitudinal axis) of all stems during the 5 years of follow-up.

**Figure 6.**
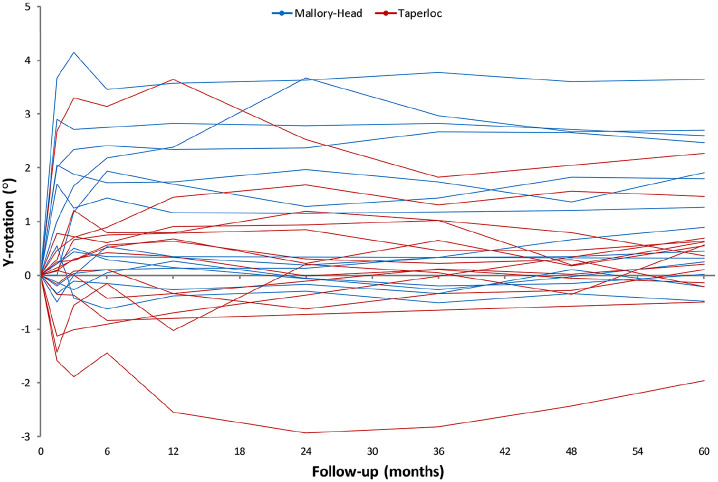
Line graphs showing the Y-rotation (i.e. internal rotation about the longitudinal axis) of all stems during the 5 years of follow-up.

On an individual level evaluation of stem migration revealed stabilisation of all stems within the first postoperative year. However, initial subsidence and retroversion varied widely. The highest subsidence for the Taperloc stems was 6.8 mm and for the Mallory-Head stems 5 mm (Figure 5). The highest retroversion was 3.6° for both stems (Figure 6).

### Clinical outcome

The postoperative HHS after 5 years of follow-up had significantly increased with an estimated mean of 44.7 points (unpaired Student’s *t*-test; 95% CI, 35.9–53.5 points; *p* < 0.001) compared to preoperative. The HHS score was not significantly different between the 2 stems throughout follow-up (LMM; *p* > 0.05) (Table 4). Between-group differences of HHS did not change significantly over time (stem type × time interaction; LMM; *p* > 0.05). As for SF-36, there were no significant differences between the 2 stems at the 2- and 5-year follow-up point (*p* > 0.05) (Table 4).

**Table 4.** Analysis of clinical outcome during 5 years of follow-up.

Outcome	Taperloc stem			Mallory-Head stem			Per protocol		
	*n*	Mean (SD)	Median (range)	*n*	Mean (SD)	Median (range)	Main effect	Group × time interaction	Prespecified time point
							*p*-Value	*p*-Value	*p*-value
HHS (min 0–max 100 points)									
Preoperative	15	42.27 (15.76)	41 (13–83)	17	44.65 (14.98)	44 (16–69)			
Week 6	16	71.19 (18.01)	77 (29–88)	16	71.63 (11.04)	75 (51–84)	0.67	0.35	
Month 3	18	79.67 (14.37)	86.5 (57–96)	17	82.29 (11.21)	84 (66–99)			
Month 6	19	82.21 (10.56)	82 (64–97)	18	90.39 (9.78)	93 (68–100)			
Year 1	18	86.39 (14.61)	91.5 (45–100)	19	91.53 (10.97)	95 (60–100)			
Year 2	19	90.63 (8.98)	94 (70–100)	16	90 (8.63)	92.5 (73–100)			0.57
Year 3	16	90.56 (8.4)	93 (69–100)	16	94.25 (3.28)	94 (89–100)			
Year 4	16	89.63 (8.87)	92 (70–99)	12	92 (7.91)	93 (73–100)			
Year 5	18	87.11 (12.87)	90 (54–100)	12	93.75 (5.71)	94 (81–100)			0.49
SF-36 (min 0–max 100 points)									
Physical component									
Preoperative	5	41.75 (14.14)	41.04 (21.68–62.84)	7	45.87 (10.44)	44.99 (28.82–59.66)			
Week 6	6	50.38 (13.1)	53.06 (24.93–57.41)	6	41.26 (9.86)	43.05 (29.45–52.83)			
Month 3	6	46.51 (9.41)	48.75 (33.59–53.12)	5	42.25 (13.8)	50.5 (19.94–52.14)			
Month 6	6	43.44 (10.53)	45.61 (24.02–51.92)	6	45.95 (9.22)	46.42 (33.82–59.19)			
Year 1	9	36.87 (13.78)	38.23 (17.98–56.6)	5	51.94 (8.07)	51.51 (39.54–59.66)			
Year 2	8	43.62 (13.75)	49.8 (20.58–52.59)	4	45.38 (5.39)	43.52 (41.21–53.27)			0.73
Year 3	9	34.69 (11.68)	33.27 (19.6–52.59)	5	39.42 (6.49)	40.45 (30–47.98)			
Year 4	7	44.95 (9.27)	50.02 (31.53–53.78)	6	43.31 (6.81)	40.15 (37.88–53.29)			
Year 5	4	37.55 (13.29)	36.95 (25.92–50.39)	6	40.71 (10.23)	38.89 (27.31–53.79)			0.62
Mental component									
Preoperative	5	48.83 (9.79)	53.53 (34.47–58.05)	7	50.53 (9.87)	49.18 (39.21–64.89)			
Week 6	6	55.21 (3.37)	56.25 (48.82–60.76)	6	50.99 (10.96)	49.25 (33.83–63.76)			
Month 3	6	55.81 (4.73)	57.34 (49.87–59.14)	5	50.03 (12.5)	50.54 (33.25–64.32)			
Month 6	6	53.01 (5.13)	52.93 (47.2–69.72)	6	49.12 (11.44)	52.15 (32.74–61.3)			
Year 1	9	53.43 (8.18)	50.69 (40.23–66.75)	5	47.83 (10.36)	49.07 (34.65–56.06)			
Year 2	8	54.32 (5.85)	53.5 (47.59–65.94)	4	47.29 (7.11)	46.94 (39.21–52.88)			0.13
Year 3	9	53.82 (8.53)	56.23 (36.95–56.9)	5	44.68 (13.59)	38.99 (33.83–47.2)			
Year 4	7	49.08 (7.95)	52.37 (39.54–59.24)	6	46.94 (10.39)	44.89 (34.34–50.48)			
Year 5	4	54.18 (4.17)	54.21 (49.07–53.65)	6	43.32 (9.69)	42.3 (38.5–62.35)			0.14

HHS, Harris Hip Score; SF-36, 36-Item Short-Form Health Survey.

### Radiographic outcome

2 stems (1 Taperloc and 1 Mallory-Head) showed non-progressive, 2-mm radiolucent lines between the stem and bone in Gruen zones 1 and 8. Interestingly, both of these stems showed high initial subsidence, 6.8 mm and 5 mm respectively. After initial subsidence, both stems stabilised and were considered not to be at risk for aseptic loosening after 5 years of follow-up.

### Survival

In 1 patient with a Mallory-Head stem, both the femoral and acetabular components were removed and a Girdlestone procedure was subsequently performed due to a deep infection of the prosthesis 3 months postoperatively. In another patient with a Taperloc stem, the liner and head were revised due to liner wear shortly after the 5-year follow-up point.

### Intention-to-treat

After randomisation and during surgery, 3 patients did not receive the allocated stem due to unfamiliarity of the surgeon with the ongoing study (Figure 1). 1 patient incorrectly received a Taperloc stem and 2 patients incorrectly received a Mallory-Head stem. Analyses of the results according to the intention-to-treat principle did not alter previous results.

## Discussion

In this randomised, clinical RSA study, hip stem migration, HHS and SF-36 were comparable between the Taperloc and Mallory-Head femoral components during 5 years of follow-up. There were no revisions for aseptic loosening and no stems were considered to be at risk for aseptic loosening. No stems showed continuous migration; that is, all stems stabilised after initial migration.

In this study, there was no significant difference in 3-dimensional migration between the 2 stems. However, the Mallory-Head stem showed more retroversion in comparison with the Taperloc stem and the variance in retroversion was larger in the Mallory-Head group. This suggests better rotational stability of the flat, wedge shaped Taperloc stem. However, rotational stability does not seem to affect subsidence; the subsidence during 5 years of follow-up as well as the subsidence rate during the first postoperative year was comparable between the 2 stems.

This is the first study comparing the migration of the Taperloc and Mallory-Head femoral components, and the first study to evaluate the migration of the Mallory Head stem. In our study the mean subsidence of the non-HA coated Taperloc stem was 1.2 mm after 2 years and this is more than the values of 0.44 mm (non-HA coated) and 0.25 mm (HA coated) subsidence at 2 year follow-up reported by Wykman and Lundberg^4^ and Bøe et al.^5^ The relatively high subsidence in our study can be explained by 2 outliers showing high initial subsidence of 4 mm and 7 mm. Furthermore, the reported subsidence in these studies might be an underestimation since the reference RSA scene was made 1 week postoperatively. Mean retroversion of 0.33° at 2-year follow-up in our study is comparable to reported values of 0.17° (HA-coated) and 0.46° (BM-coated).^5^ Flatøy et al.^6^ reported the 5-year results of the same study published earlier by Bøe et al.,^5^ showing initial subsidence up to 10.4 mm with subsequent stabilization of all stems. Nebergall et al.^7^ reported comparable results to our study with a similar non-HA coated Taperloc stem, showing initial migration up to 9.3 mm with subsequent stabilisation of all stems and a median subsidence of 0.03 mm after 5 years of follow-up.^7^

There was a high variation in initial migration for both the Taperloc and the Mallory-Head stem. The Taperloc stem showed up to 7 mm of subsidence and the Mallory-Head stem showed up to 5 mm of subsidence. Both stems showed initial retroversion of about 3°. For all stems, initial migration occurred during the first 3 postoperative months. Stems showing little initial migration quickly stabilised, while stems showing large initial migration took up to 2 years to stabilise. After the second postoperative year, all stems had stabilised. This suggests that high initial migration is acceptable as long as the stem does not continue to migrate and ultimately stabilises. All implants showing high initial subsidence also showed high initial rotation into retroversion. 2 of the stems showing high initial migration, showed non-progressive radiolucencies of 2 mm. In the other stems, there were no radiolucencies present.

This study shows that it can take up to 2 years before the stem stabilises. Several studies with cemented stems have shown that high initial migration is predictive of late aseptic loosening.^8,9^ Some of these studies have shown that high initial migration during the first postoperative year is already predictive of late aseptic loosening.^22^ This study, however, demonstrates that it can take up to 2 years before an uncemented implant stabilises without being at risk of aseptic loosening after 4 years of follow-up. The failure mechanism of uncemented stems might therefore be different from cemented stems.^23^

We should also consider some limitations. Firstly, this was one of the first RSA studies performed at our institution; hence there was little experience with the procedure of placing tantalum markers in the periprosthetic bone. Therefore, 8 patients had to be excluded due to marker problems. However, no patients were lost to follow-up. Secondly, there was no difference in stem morphology in terms of Canal Flair Index or Dorr classification. Different stem morphologies might favour one femoral stem design over another. Unfortunately, this study was not powered to make such recommendations. Thirdly, this trial was not prospectively registered in an ICMJE approved registry. At the time this study was designed, registration of trials had not yet been established.

In conclusion, this study confirms the excellent clinical and survival results of the Taperloc and Mallory-Head stems provided by survival studies. Both stems are safe choices in total hip arthroplasty. The Taperloc provides better initial rotational stability but the clinical benefit of this has not been proven.
